# Examining Differences in Utilization of the Ontario eConsult Service in Rural Versus Urban Settings: A Retrospective Cross-Sectional Analysis

**DOI:** 10.1177/21501319251354830

**Published:** 2025-07-16

**Authors:** Clare Liddy, Sheena Guglani, Nikhat Nawar, Erin Keely

**Affiliations:** 1 C. T. Lamont Primary Healthcare Research Centre, Bruyere Health Research Institute, Ottawa, ON, Canada; 2 Department of Family Medicine, University of Ottawa, Ottawa, ON, Canada; 3 eConsult Centre of Excellence, The Ottawa Hospital, Ottawa, ON, Canada; 4 Department of Medicine, University of Ottawa, ON, Canada; 5 Department of Medicine, The Ottawa Hospital, Ottawa, ON, Canada

**Keywords:** eConsult, electronic consultation, equity of access, primary care, rural, specialist, urban

## Abstract

**Introduction:**

We conducted a retrospective, cross-sectional analysis exploring patterns of usage and outcomes from urban vs. rural eConsults to examine eConsult’s impact on equity of access in rural Ontario, Canada. Patients living in rural regions face many barriers in accessing specialist care. The Ontario eConsult Service connects primary care providers (PCP) with specialists regardless of geographical location, improving equity of access.

**Methods:**

We included all Ontario eConsult cases submitted between January 1 and December 31, 2021. Usage data collected automatically by the service and responses to a mandatory closeout survey were analyzed using descriptive statistics. Cases were identified as rural using the forward sorting area of the PCP’s primary practice.

**Results:**

Of the 72,948 cases submitted during the study period, 7550 were coded rural. Usage among rural PCPs was most frequent in Ontario Health North East (1.78 eConsult cases/1000 residents) and Ontario Health North West (1.64). Rural and urban eConsult cases had the same top 5 most frequently requested specialties. Both groups had median response times of 1.0 days, reported time billed of 15 min, and cost per case of $50.

**Conclusions:**

PCPs in rural and urban regions use eConsult with equal frequency and had similar usage patterns and outcomes.

## Introduction

Patients living in rural areas often face greater challenges in accessing timely healthcare services.^1,2^ Scarcer services, fewer healthcare professionals, greater distances, limited public transit, and lower internet connectivity are all factors that impede rural patients’ access to care.^3^ The problem is particularly acute for specialist care, as specialist practitioners are more likely to practice in urban areas with large populations.^2^ This leads to poorer health outcomes in rural areas, including increased infant mortality, lower life expectancies, and higher incidence of cardiovascular disease.^4,5^

Telemedicine can help bridge this gap by allowing rural patients to access expert advice from healthcare professionals without the burden of travel. Digital healthcare services encompass various approaches to provide remote consultation and diagnosis, often leading to shorter waiting times.^6^ They can be synchronous, such as phone or video consultations, or asynchronous text-based messaging systems. Some telehealth services involve a patient speaking directly with their healthcare provider, whereas others connect healthcare professionals to facilitate discussion about individual patient cases. Electronic consultation (eConsult) services are commonly defined as secure digital platforms that support asynchronous communication between clinicians.^7,8^ The most highly-studied eConsult services are in the United States (with the highest number of identified services, most notably the Veteran Affairs model) and Canada (most notably the Champlain BASE^TM^ model).^7,9^ Many other eConsult systems exist worldwide, with ongoing expansion to new countries.^8^

The Ontario eConsult Service is an established program, accessible by all primary care providers (PCPs) across Ontario.^10^ It uses the Champlain BASE^TM^ model, which is highly scalable,^11,12^ cost-effective,^13^ and has a strong record of success in Ontario and several other provinces.^10,14^ This model uses a secure web-based platform to facilitate asynchronous communication between healthcare providers, allowing PCPs to access timely advice from specialists.^10^ Ontario is Canada’s most populous province and its residents live in a wide variety of settings ranging from highly urban to highly remote.^15^ Thus, the Ontario eConsult Service dataset provides a unique opportunity for comparison between urban and rural settings.

Two earlier studies explored the use of eConsult for improving healthcare access for patients living in Nunavut and those with complex circumstances in rural areas.^16,17^ Similar programs for facilitating electronic access to specialists are being evaluated in other rural areas, including several regions in the United States^18,19^ and Australia.^20,21^ This study builds on that work, examining provincial-level eConsult data in Ontario.

Our study has 3 main objectives: (1) to determine the proportion of urban vs. rural eConsult cases in each Ontario Health (OH) Region; (2) to compare patterns of usage and outcomes from urban vs. rural eConsult cases in Ontario; and (3) explore the impact of eConsult on equity of access in rural Ontario across different health regions.

## Methods

### Design

We conducted a retrospective, cross-sectional descriptive analysis of eConsult cases submitted to Ontario eConsult due to the availability of high-quality utilization data routinely collected and cleaned up by the service.

### Setting

Cases were examined from all 6 Ontario Health regions. The North East (population: 0.6 million) and North West (0.2 million) regions are largely rural communities, whereas the Toronto region (1.4 million) is highly urban and the Central (5 million), East (3.7 million), and West (4 million) regions are a mixture of urban and rural communities.^15^ This setting was selected based on the availability of high-quality data for a large number of people in diverse settings, including rural and urban communities.

### Participants

All eConsult cases submitted in Ontario between January 1st, 2021 and December 31st, 2021 were included in the analysis to examine the service throughout a typical year; thus, no power calculation was required. Cases were excluded if any relevant data was missing (e.g., postal code). Participants (i.e., healthcare professionals participating in eConsults) were not identified/selected, so no exclusion/inclusion criteria were used.

### Intervention

To use eConsult in Ontario, PCPs log into the application using any device with an internet browser and enter their question, attaching any relevant files (e.g., images, test results). They select a specialty group from a menu of 137 specialties (BASE^TM^ model) or a specific specialist (direct to specialist model) and submit.^22^ A case assigner allocates the eConsult case to a specialist in the chosen group based on availability. The specialist is expected to respond to the question within 1 week, providing advice on care, recommendation for a referral, or a request for further information. Discussion can continue until the PCP chooses to close the case.

### Data Collection

We used data routinely collected during the eConsult process, including utilization data (e.g., specialty selected, time between question and response, specialists’ self-reported time spent on the response) and PCP responses to a mandatory closeout survey, which they complete upon conclusion of each eConsult. The survey includes questions asking PCPs to assess whether the service confirmed an existing opinion, provided new or additional information, or was not particularly useful, and whether a referral was originally needed/ultimately completed.

Response time was defined as the time between the eConsult question and the first response. Cost per case was determined based on the billing cost ($200 per hour, pro-rated) and time spent per case, in minutes.

To identify whether a PCP’s practice was rural, we used the forward sortation area (FSA of the primary organization for each requesting provider. FSAs with 0 as the second character were identified as rural and values 1 to 9 were identified as urban. This method is used by Canada Post to identify rural delivery areas.^23,24^ Although the postal code does not specify an exact location, it is a broadly effective means of identifying areas that are outside of urban areas and serviced by rural route drivers and/or postal outlets.^24^

### Data Verification and Analysis

Data cleanup and verification were done through a predefined process, including removing duplicates, identifying missing fields, correcting misspellings, and ensuring data were in the correct formats.

Quantitative data analysis was completed in Excel using descriptive statistics to describe the dataset. For each health region, we calculated counts of eConsult cases, number of cases per 1000 residents, and percentage of urban vs. rural cases. We calculated average and median cases submitted by the PCPs. We combined overlapping specialty groups (e.g., pediatric cardiology/cardiology) and alternate spellings (e.g., anesthesiology/anesthesiology) before calculating percentages submitted to each group. We calculated medians for response time, billing time, and cost per case, and calculated percentages for each survey response. No other statistical tests were done, and no other software was used.

## Results

**Figure 1.**
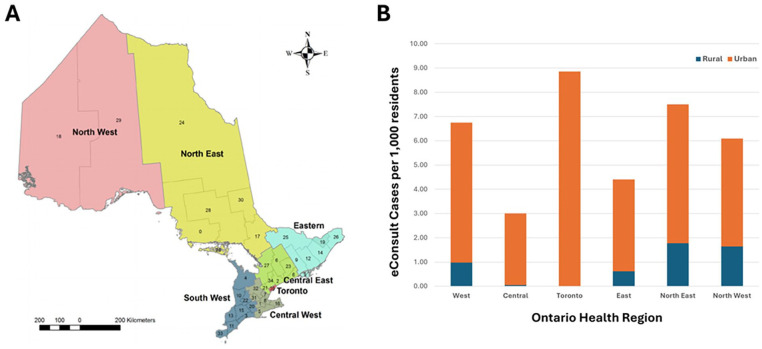
(A) Map of the Ontario Health Regions with local health units numbered 1 to 35. Reproduced with permission from *BMC Public Health.*^25^ (B) Urban and rural eConsult cases per 1000 residents in each health region.

In total, 72,948 eConsult cases were submitted in Ontario in 2021. Of these, 7550 eConsult cases submitted by 574 PCPs were coded rural (10%) and 64,469 eConsult cases submitted by 4872 PCPs were coded urban (88%); the remaining cases were missing postal code data (929 cases) or health region (27 cases) and were excluded from analysis. Urban PCPs submitted an average of 13.23 cases each and rural PCPs submitted an average of 13.15 cases each; the median number of submitted cases was 6 for both groups. Usage by rural PCPs varied by Ontario Health (OH) region. When total eConsult cases were adjusted for the population of each health region, OH North East had the highest number of eConsult cases placed by rural PCPs, with 1.78 rural eConsult cases completed per 1000 residents, followed by OH North West (1.64), OH West (0.98), OH East (0.62), and OH Central (0.05) (Figure 1). In the same regions, urban eConsult rates were 5.72 per 1,000 residents in OH North East, 4.45 in OH North West, 5.77 in OH West, 3.79 in OH East, 2.95 in OH Central, and 8.86 in OH Toronto. The regions with the highest proportion of rural eConsult cases were OH North East (23.01%) and OH North West (26.5%), followed by OH West (14.35%), OH East (13.89%), OH Central (1.73%), and OH Toronto (none).

The most frequent specialty services selected by rural PCPs were the following: dermatology (18%), obstetrics/gynecology (7%), hematology (7%), allergy and clinical immunology (7%), and endocrinology (6%); additional specialties are shown in Table 1. PCPs in urban areas requested advice most frequently from dermatology (14%), hematology (8%), obstetrics/gynecology (7%), allergy and clinical immunology (7%), and endocrinology (6%). Both groups had median response times of 1.0 days, reported time billed of 15 min, and cost per case of $50.

**Table 1.** Specialty Distribution in Rural and Urban eConsult Cases.

Grouped specialty	URBAN cases	% of URBAN cases	RURAL cases	% of RURAL cases
Allergy and Clinical Immunology	4713	7.44	491	6.60
Anatomical Pathology	1	0.00	0	0
Anesthesiology	192	0.30	30	0.40
Cardiac Surgery	68	0.11	7	0.09
Cardiology	2789	4.40	305	4.10
Cardiothoracic Surgery	3	0.00	0	0.00
Clinical Pharmacology	162	0.26	13	0.17
Colorectal Surgery	9	0.01	0	0.00
Community Medicine	183	0.29	19	0.26
Critical Care Medicine	178	0.28	14	0.19
Dentistry	24	0.04	1	0.01
Dermatology	8935	14.11	1336	17.95
Emergency Medicine	1	0.00	0	0.00
Endocrinology	4079	6.44	444	5.96
ENT	1058	1.67	138	1.85
Family Medicine	1099	1.74	131	1.76
Gastroenterology	2553	4.03	260	3.49
General Surgery	530	0.84	57	0.77
Genetics	425	0.67	36	0.48
Geriatrics	211	0.33	43	0.58
Gynecologic Reproductive Endocrinology and Infertility	41	0.06	3	0.04
Hematology	4986	7.87	517	6.95
Infectious Disease	2446	3.86	221	2.97
Internal medicine	3640	5.75	418	5.62
Medical Biochemistry	4	0.01	0	0.00
Microbiology	25	0.04	2	0.03
Neonatal-Perinatal Medicine	34	0.05	4	0.05
Nephrology	1094	1.73	91	1.22
Neurology	3749	5.92	405	5.44
Neurosurgery	694	1.10	83	1.11
OBSGYN	4377	6.91	528	7.09
Oncology	528	0.83	56	0.75
Ophthalmology	427	0.67	52	0.70
Orthopedic Surgery	1723	2.72	245	3.29
Other	165	0.26	16	0.21
Pain Medicine	32	0.05	5	0.07
Palliative Medicine	16	0.03	0	0.00
Pediatric Development	6	0.01	1	0.01
Pediatrics	2326	3.67	310	4.16
Physical Medicine & Rehabilitation	140	0.22	18	0.24
Plastic Surgery	238	0.38	27	0.36
Psychiatry	3419	5.40	408	5.48
Public Health	282	0.45	28	0.38
Radiology	565	0.89	28	0.38
Respirology	672	1.06	93	1.25
Rheumatology	1788	2.82	235	3.16
Thoracic Surgery	89	0.14	11	0.15
Urology	2054	3.24	239	3.21
Vascular Surgery	564	0.89	75	1.01

**Figure 2.**
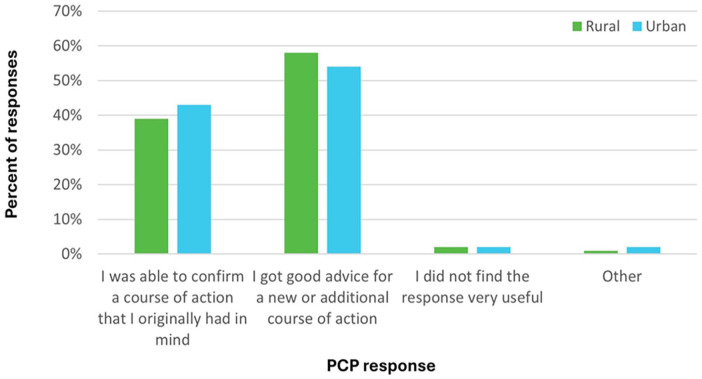
Responses to survey question assessing whether eConsult confirmed an existing course of action or provided a new or additional course of action, divided by rurality.

**Figure 3.**
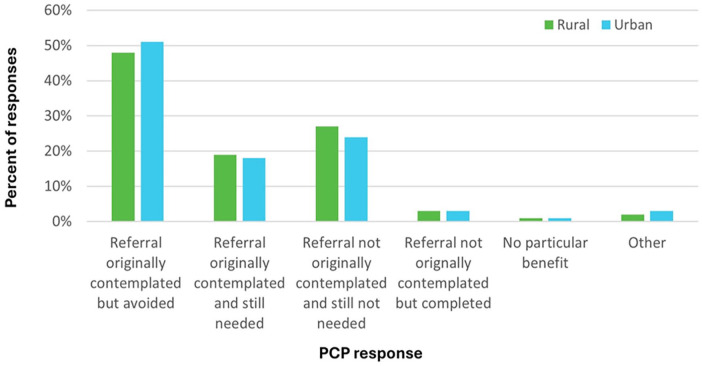
Responses to survey question assessing eConsult’s impact on decision to refer, divided by rurality.

Survey outcomes were similar between rural and urban eConsult groups. Rural PCPs were slightly more likely to report receiving advice for a new or additional course of action (58% rural vs. 54% urban; Figure 2), while urban PCPs were slightly more likely to report a referral as “originally contemplated but avoided” based on the response received from the eConsult (48% rural vs. 51% urban; Figure 3). After the eConsult, rural and urban PCPs were equally likely to complete a referral, either one that was originally planned (19% rural; 18% urban) or one that was not originally planned (3% for both groups).

## Discussion

Overall, our study found very similar patterns of eConsult usage between rural and urban PCPs, with the same top 5 specialties selected in the 2 groups. The Ontario Health regions differed in their proportions of rural vs. urban eConsult cases completed, which may be due to differences in rurality between the regions, as rural and urban PCPs placed similar numbers of eConsult cases per PCP; however, we could not directly assess patient rurality in this study to confirm. Case outcomes were also very similar for both groups, with nearly identical response times, case costs, and survey outcomes. These similarities suggest that patients in rural and urban regions receive equivalent service from eConsult regardless of their location, making it an important driver of equity in access to specialist care.

To explore eConsult equity of access, we compared how frequently eConsult services were used in urban and rural environments. Cases submitted by PCPs in rural areas accounted for slightly over 10% of the total case volume during the study period, which was similar to the proportion of rural PCPs in our study. A 2018 study found that 9.3% of Ontario’s family physicians practice in rural areas.^26^ Although not all PCPs are family physicians, the similarity between these numbers suggests that rural and urban PCPs are using eConsult at a roughly similar frequency. However, approximately 17% of Ontarians live in rural regions,^27^ so rural patients may be underrepresented in the overall proportion of eConsult cases. While different methods were used to calculate rurality in our study, making it difficult to compare directly, the discrepancy is large enough to suggest a possible gap in equity of access. This gap could be caused by differences in access to primary care. Since patients cannot use eConsult directly, but instead must rely on a PCP accessing the service on their behalf, usage is by necessity limited to patients with some access to primary care. Rural areas have been hit harder by the critical shortage of healthcare workers in Ontario, with rural doctors carrying heavy patient loads and rural Ontarians losing access to primary care at 4 times the rate of those in urban areas.^28^ At least 376,000 rural Ontario residents had no access to primary care in 2022, and the number is rising due to significant difficulty recruiting new family doctors to replace those retiring.^28^ Furthermore, rural patients who need to travel to an urban center to access healthcare would be classified as “urban” in this study. The eConsult service would be particularly useful for these patients and for all rural residents, who are more likely to face extensive time and cost barriers when traveling to specialist appointments.

To support equitable access to healthcare for rural and remote communities, the College of Family Physicians of Canada and the Society of Rural Physicians of Canada convened a taskforce for developing a Rural Roadmap to Action.^29^ The taskforce proposed various actions, including “establish practice models that provide rural and Indigenous communities with timely access to quality health care that is responsive to their needs.” The taskforce emphasized the frustrations that rural PCPs and their patients experience when attempting to access specialist care, and recommended developing “specific resources, infrastructure, and networks of care within local and regional health authorities to address access issues.”^29^ We have shown here that eConsult can be one of those resources to support equitable access in rural areas.

***Bias and Limitations*:** Our study has several areas of potential bias and limitations. A significant limitation is that our method of classifying rurality may misclassify some patients. While using the second character of the FSW is a broadly effective means to identify an area as rural, different identification methods may class regions differently. For example, Statistics Canada uses population density to classify areas as “rural” in the census population, and some of those areas are not considered “rural delivery areas” by Canada Post.^24^ This makes it difficult to compare our findings with studies that use different definitions of rural. Furthermore, provider locations rather than patient home addresses were used to determine rurality. Because many patients living in rural regions must travel long distances to receive care from urban providers, the rurality of the providers and patients may differ. This is likely to cause underestimation of rural patient access. Some eConsult cases (1%) were excluded from analysis due to missing data, which could bias the results. While our study identified a few small differences in survey outcomes between rural and urban eConsult cases, we were unable to assess the potential differences in outcomes at a patient level. In addition, our study analysis was done via spreadsheet rather than in a scripted environment, which may affect reproducibility. There is also a selection bias, as eConsult use is voluntary, and there may be differences between the PCPs that choose to use it and those that do not (e.g., comfort level with digital tools).

Further research is needed to complete a taxonomy of rural eConsult cases and determine whether rural and urban PCPs ask different types of questions, as our study did not look at the content of PCP questions or specialist responses. Future studies using health administrative data or qualitative interviews would be helpful to directly examine the experiences and outcomes of rural patients.

## Conclusion

PCPs in rural and urban regions submit equal numbers of eConsult cases, and the patterns and outcomes of usage are very similar between the 2 groups. However, the proportion of rural eConsult cases is lower than the proportion of rural residents, suggesting there may be a difference in access to care. The service’s ability to connect PCPs with advice from a full menu of specialist services from other locations increases equity of access for rural patients, who otherwise must often travel great distances for specialist care. Furthermore, the eConsult service provides answers more rapidly than traditional referrals. This would be expected to have a positive effect on individual patient outcomes, as well as overall rural population health. Thus, eConsult is an important tool for rural providers to ensure their patients have equitable access to specialist advice. To improve equity of access, rural areas where eConsult is currently underutilized could be targeted for enhanced promotion of eConsult services.
